# Melamine-Assisted Thermal Activation Method for Vacancy-Rich ZnO: Calcination Effects on Microstructure and Photocatalytic Properties

**DOI:** 10.3390/molecules28145329

**Published:** 2023-07-11

**Authors:** Weiwei Wang, Lin Lv, Changfeng Wang, Jiao Li

**Affiliations:** 1 School of Materials Science and Engineering, Shandong University of Technology, Zibo 255049, China; 2 Shandong LinJia New Material Technology Co., Ltd., Zibo 255049, China

**Keywords:** zinc oxide, oxygen vacancy, melamine assist, oriented growth, photocatalysis

## Abstract

Defect engineering is considered an effective method to adjust the photocatalytic properties of materials. In this work, we synthesized the vacancy-rich ZnO rods with (100) planes via the melamine-assisted thermal activation method. A high concentration of oxygen vacancies was successfully introduced into non-polar oriented ZnO rods by calcination. The effect of oxygen vacancy on the photocatalytic properties of non-polar-oriented ZnO rods was investigated. Raman and XPS spectra revealed the formation of oxygen vacancies in the ZnO. The results showed that the growth habit and defects in ZnO can be controlled by changing the ratio of ZnO to melamine. The higher ratio of ZnO to melamine led to more amounts of (100) planes and oxygen vacancies in ZnO, and it reached the highest when the ratio was 1.2:1. When the ratio was 1.2:1, ZnO exhibited a high methyl orange degradation rate (95.8%). The differences in oxygen vacancy concentration and non-polar planes were responsible for the improvement in photocatalytic performance. ZnO exhibited good stability and regeneration capacity. After recycling four times, the degradation rate was still at 92%. Using the same method, vacancy-rich α-Fe_2_O_3_ was obtained. This work could offer a new and simple strategy for designing a photocatalyst with oxygen vacancies.

## 1. Introduction

Calcination is an effective way to prepare materials with good crystallinity and properties. Compared with other preparation methods, calcination has the advantages of simple operation, lower requirements of equipment, increases in molecular/ion activity and the provision of a special environment to form defects, thus affecting the crystal growth kinetics. All of these advantages are beneficial to obtain materials with different morphologies, structures and properties. Upon calcination, a systematic morphology, structure and property can be monitored by changing temperature, time, or atmosphere, or adding additives [1,2,3]. For example, with an increase in calcination temperature (from 300 °C to 400 °C), Cu^II^_0.4_Fe^II^_0.6_Fe^III^_2_O_4_ nanomaterials changed from an irregular morphology to needle-like structure, and the photocatalytic performance was significantly improved [2]. During the calcination process, less-reactive monoclinic α-spodumene was converted to more-reactive tetragonal β-spodumene, which resulted in a higher lithium grade and recovery [3]. 

Zinc oxide (ZnO) is a unique nontoxic material with high photocatalytic activity and chemical stability [4], which shows strong face-dependent and defect-dependent photocatalytic activity [5,6]. Many researches have focused on how to form specified faces and defects in the structure of ZnO to improve its photocatalytic performance. Calcination is an effective way to obtained ZnO with defects and orientation growth. For example, defects such as oxygen vacancies were generated by annealing ZnO under oxygen-deficient conditions or in a reductive environment [7]. The growth habit of ZnO could be modified during the calcination process by introducing plasma [8], electrospinning [9], or additives [10]. Among this research, ZnO with polar (001) planes was easily obtained. However, it has been reported that ZnO with non-polar planes could exhibit higher stability and properties. For example, ZnO with non-polar (100) planes showed higher gas-sensing properties compared to that with polar (001) planes [11]. Therefore, the performance of ZnO is expected to improve by combining defect engineering with orientation growth. Though a few studies reported the formation of defects in non-polar-oriented ZnO rods, a more straightforward and efficient way to create defects is still urgently needed [12].

In this work, non-polar-oriented ZnO rods with oxygen vacancies created by a melamine-assisted thermal activation method were obtained. The growth habit and defects in ZnO were controlled by changing the ratio of ZnO to melamine. Simply calcining ZnO with melamine introduced oxygen vacancies in the structure of ZnO and realized the orientation growth of ZnO rods. The effect of oxygen vacancies on the photocatalytic property of non-polar-oriented ZnO rods was investigated. We also prepared vacancy-rich α-Fe_2_O_3_ using the same method.

## 2. Results and Discussion

### 2.1. Structure and Morphology Characterization

**Figure 1:**
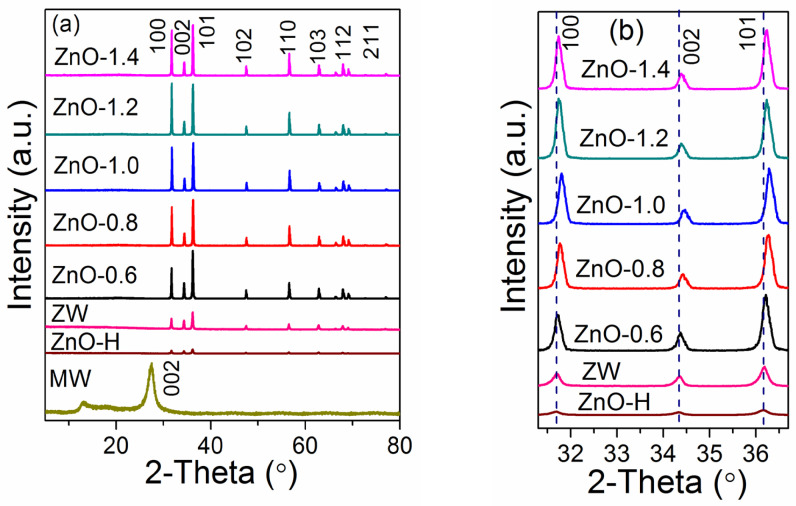
(**a**) XRD patterns of MW, ZnO-H, ZW and ZnO-X, and (**b**) the enlarged XRD patterns of ZnO-H, ZW and ZnO-X in the region of 2θ = 31–37°.

The structures of samples were analyzed by XRD patterns (Figure 1). A single phase of ZnO with a hexagonal structure was obtained after reaction at 120 °C (sample ZnO-H, Figure 1a, JCPDS No. 36-1451). For comparison, melamine was calcined at 520 °C for 4 h, which resulted in the formation of g-C_3_N_4_ with a hexagonal phase (sample MW, JCPDS file No. 87-1526). After calcining ZnO-H at 520 °C with and without melamine (labeled as sample ZnO-X and sample ZW, respectively), all samples showed the same hexagonal structure of ZnO (Figure 1a), but the crystallinity increased. After calcining ZnO with melamine, no diffraction peaks for g-C_3_N_4_ were detected, which confirmed that there were no significant changes in the crystal structure. As the ratio of ZnO to melamine increased, the peak intensity increased gradually, indicating an enhancement in the crystallinity. In general, materials with higher crystallinity show better photocatalytic activity and stability since the increase in crystallinity could decrease the number of recombination centers for photoinduced charges, leading to efficient charge migration [13]. Therefore, ZnO-X with higher crystallinity is expected to show better photocatalytic activity.

Furthermore, the magnified XRD patterns (Figure 1b) revealed that the diffraction peaks of ZnO in the sample ZnO-X shifted toward a higher scattering angle in comparison with ZW and ZnO-H, which was attributed to the formation of vacancies [14]. It can be further proved by the change in lattice parameters. Based on the equations (Equations (1)–(3), which were derived from Equations (S1) and (S2)), the lattice parameters (*a* and *c*) and the cell unit volume (*V*) were calculated using the lattice spacing values of (100) and (002) planes. The values obtained are summarized in Table 1. The slight decrease in the lattice parameters was observed as the ratio increased (Table 1 and Figure S1). Compared to ZW, the peak intensity and the tendency of oriented growth of ZnO rods in ZnO-X were increased (Figure S1), indicating that the addition of melamine changed the growth habit of ZnO rods. The relatively high intensity of (100) peaks was obtained when the ZnO/melamine ratio was 1.2:1.

**Figure 2:**
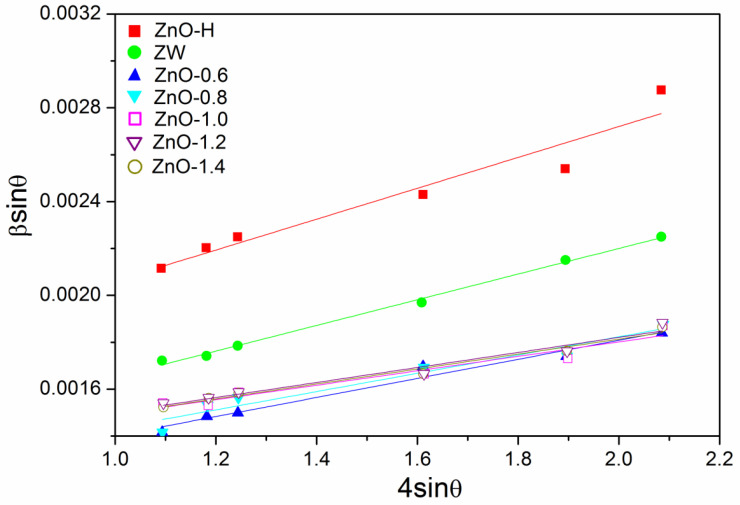
Plots of *β*cos*θ* versus 4sin*θ* for ZnO-H, ZW and ZnO-X.

The average crystallite sizes of samples were calculated using Scherrer’s formula (Equation (4)) and the Williamson–Hall model (W-H model, Equations (5) and (6), Figure 2) [15].

(1) $$a=\lambda /(\sqrt{3}\sin\theta_{\left(100\right)})$$

*c* = λ/sin*θ*_(002)_ (2)

(3) $$V=\sqrt{3}a^{2}c/2$$

*D* = 0.9λ/*β*_D_cos*θ* (4)

*β*cos*θ* = 0.9λ/*D* + 4*ε*sin*θ* (5)

*β* = *β*_D_ + *β_ε_* (6)

where λ, *θ*, *D*, *β*_D_, *ε* and *β_ε_* represent the wavelength of the CuKa radiation, Bragg angle, crystallite size, full-width at the half maximum intensity (FWHM) of the diffraction line, strain and the strain-induced broadening of the diffraction line. The values of *ε* were also obtained from the slope of *β*cos*θ*-4sin*θ* plots (Table 1). It can be seen that the sizes of samples calculated with Scherrer’s formula are smaller than those of the W-H model (Table 1). The reason is that the strain-induced broadening effect is excluded in Scherrer’s formula. After calcining ZnO with or without melamine, the values of ε in samples decreased and the sizes of ZnO increased, confirming the improvement of crystallinity of ZnO.

**Figure 3:**
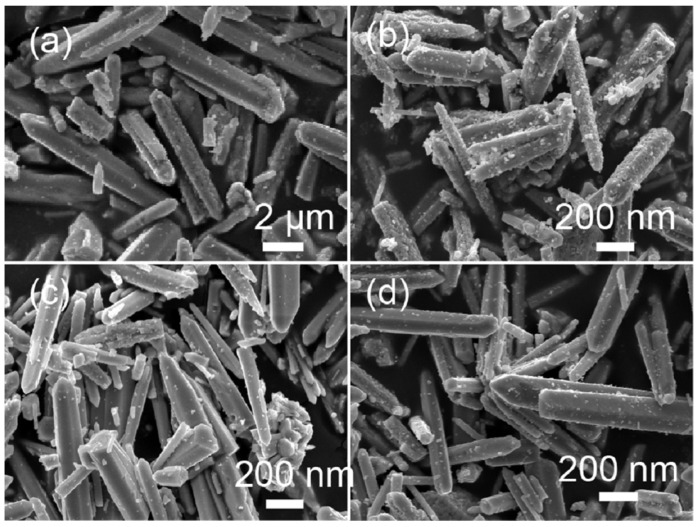
SEM images of (**a**) ZW, (**b**) ZnO-0.8, (**c**) ZnO-1.0, and (**d**) ZnO-1.2.

**Figure 4:**
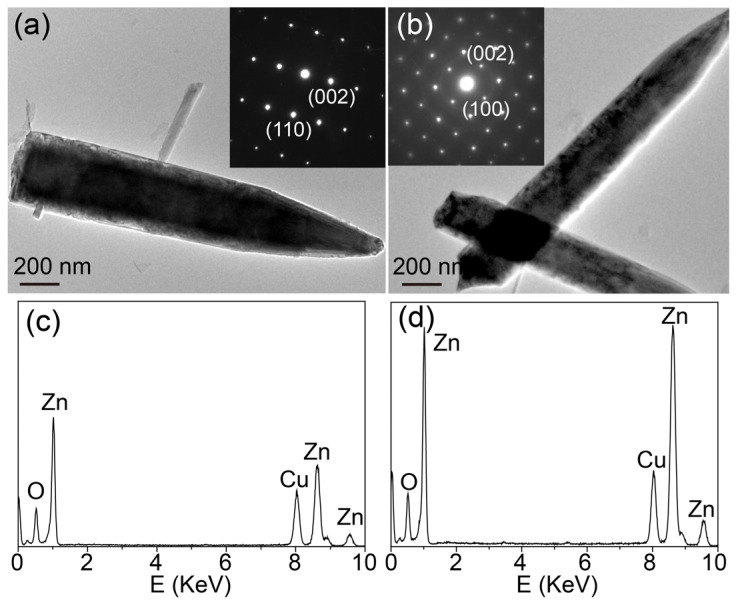
TEM images of (**a**) ZW and (**b**) ZnO-1.2, insets: corresponding SAED patterns, EDS spectra of (**c**) ZW and (**d**) ZnO-1.2.

Sample ZW exhibited a hexagonal rod-like shape with a size of about 200 nm (Figure 3a). When annealing ZnO-H with melamine in air, all ZnO-X showed a similar hexagonal rod-like shape (Figure 3b–d), while the sizes of the ZnO rods decreased. TEM, SAED and EDS analyses were employed to investigate the microstructures of ZnO calcined with and without melamine (Figure 4). Both ZW and ZnO-1.2 showed a rod-like shape. The SAED patterns of a single rod revealed the crystalline nature of ZW and ZnO-1.2 (insets in Figure 4a,b). The SAED pattern of ZnO-1.2 could be indexed to the (002) and (100) reflection of ZnO, while that for ZW was indexed to the (002) and (110) reflection. EDS spectra also confirmed that these rods were mainly composed of Zn and O elements (the Cu element came from the TEM copper grid of the sample holder, Figure 4c,d). This result demonstrated that calcining ZnO with melamine changed the growth habit of ZnO, which was in accordance with XRD analyses.

### 2.2. XPS and Raman Spectroscopy Results: Formation of Oxygen Vacancy

**Figure 5:**
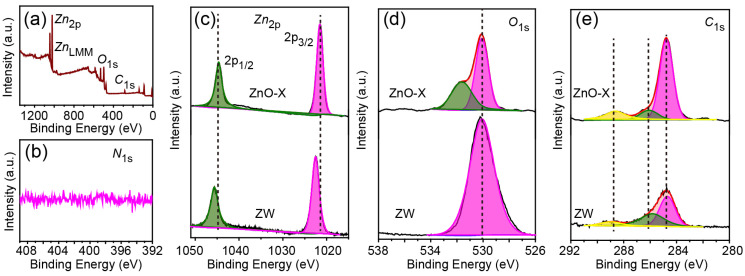
(**a**) the wide-scan XPS spectrum, the high-resolution XPS spectra of (**b**) N 1*s*, (**c**) Zn 2*p*, (**d**) O 1*s* and (**e**) C 1*s*.

XPS spectra were used to characterize the surface chemical composition of ZnO and ZnO-X. The wide-scan XPS spectrum of ZnO-X revealed that ZnO-X was mainly composed of Zn, O and C elements (Figure 5a). No obvious peak for N was observed (Figure 5b), which confirmed that no g-C_3_N_4_ was obtained after calcining ZnO with melamine.

The high-resolution XPS spectrum of Zn 2*p* for ZnO-X displayed two peaks at 1021.5 eV and 1044.6 eV, corresponding to the Zn 2*p*_3/2_ and Zn 2*p*_1/2_ of ZnO [16]. The difference in binding energy between Zn 2*p*_1/2_ and Zn 2*p*_3/2_ states was 23.1 eV, which matched well with the standard value (23.0 eV) (Figure 5c). For ZW, two peaks appeared at around 1022.5 eV (Zn 2*p*_3/2_) and 1045.6 eV (Zn 2*p*_1/2_) with a similar energy distance (23.1 eV). It is worth noting that the shift of the peak for ZnO-X toward lower binding energy confirmed an increase in the electron density of the Zn^2+^ species, which resulted from the formation of oxygen vacancies in ZnO-X [17].

The high-resolution XPS spectrum of O 1*s* for ZnO-X was fitted with two peaks, labeled as O_L_ and O_V_ and located at 530.15 eV and 531.71 eV, respectively (Figure 5d). The O_L_ peak and O_V_ peak can be assigned to the lattice oxygen of ZnO and oxygen ions in the oxygen-deficient regions caused by oxygen vacancies [18,19]. However, the XPS spectrum for ZW was only fitted with one peak at 530.15 eV (O_L_), and no peak for O_V_ was observed. It is believed that the peak area ratio of O_V_/(O_L_ + O_V_) reveals the concentration of oxygen vacancies [16,18]. O_V_/(O_L_ + O_V_) was 38.5% for ZnO-X, which suggested the formation of oxygen vacancies in ZnO-X. The Zn/O atomic ratio in ZnO could be larger than the stoichiometric value (Zn:O = 1:1), owing to the formation of interstitial ions or ions vacancies [18]. According to the XPS analyses, the Zn/O_L_ atomic ratio was 1.19:1 for ZnO-X, and oxygen vacancies in the surface of ZnO-X were 16%, which confirmed the formation of oxygen vacancies for ZnO-X, similar with reported values [20,21].

The high resolution XPS spectra of C 1*s* (Figure 5e) were fitted with three peaks, located at 284.8 eV, 286.4 eV and 288.6 eV for ZnO-X as well as 284.6 eV, 285.9 eV and 288.5 eV for ZW, which were attributed to C–C, C–O and C=O, respectively [19,22]. Compared to ZW, the peak of C 1*s* in ZnO-X shifted slightly toward higher binding energy. The peak intensity ratio of 1288.6 eV/(1284.8 eV + 1286.4 eV + 1288.6 eV) for ZnO-X (16.42%) was larger than that of ZW (8.96%). The light yellow color of ZnO-X further indicated the presence of C in ZnO-X after ZnO-H was calcined with melamine.

**Figure 6:**
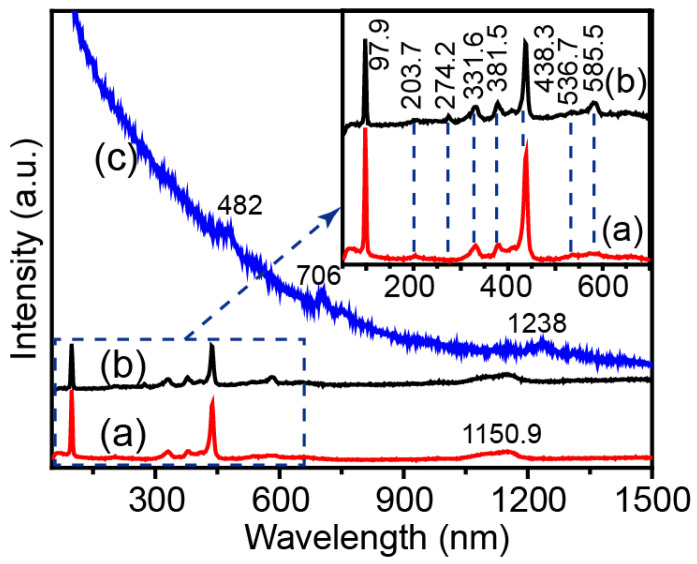
Raman spectra of (a) ZW, (b) ZnO-X and (c) MW. Inset is the enlarged Raman spectra of ZW and ZnO-X.

Raman spectroscopy was used to study the molecular vibration and chemical structure, which revealed the defects in ZnO. Compared to MW (Figure 6), no peaks of g-C_3_N_4_ were observed for ZnO-X. Several peaks for ZnO were observed (Figure 4a,b). The peaks at 97.9 cm^−1^ and 438.3 cm^−1^ were attributed to the E_2_ vibration mode of ZnO with a hexagonal structure [16]. The peaks at 203.7 cm^−1^, 331.6 cm^−1^, 381.5 cm^−1^ and 1150.9 cm^−1^ were assigned to the molecular vibration arising from zero-boundary phonons 2-TA(M), 2E_2_, A_1_ (TO) and 2E_1_(LO) modes, respectively [22]. The peak around 585.5 cm^−1^ was assigned to the E_1_(LO) mode, owing to the contribution of oxygen vacancies in ZnO. Compared to ZW, ZnO-X showed relatively higher intensity of the peak located at 585.5 cm^−1^, which revealed that ZnO-X has more defects than ZW.

### 2.3. Diffuse Reflectance Spectroscopy Results: Band Position and Optical Property

**Figure 7:**
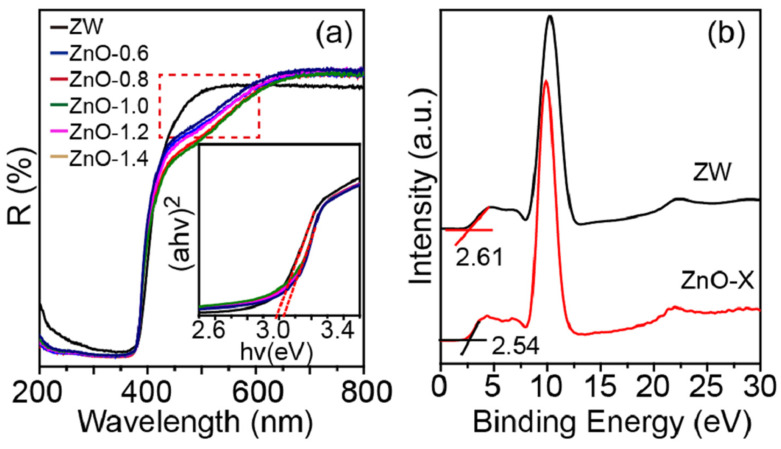
(**a**) UV-Vis diffuse reflectance spectra of ZW and ZnO-X; inset is the corresponding (*αhν*)^2^-*hν* curves, (**b**) XPS valence band spectra of ZW and ZnO-1.2.

Figure 7a shows the room-temperature UV-Vis diffuse reflectance spectra of ZW and ZnO-X. The increasing adsorption for ZnO-X in the region between 450–600 nm shows the enhanced adsorption properties of ZnO-X (as indicated by a square in Figure 7a), which was related to the surface defects [18]. According to the Tauc’s model (Equation (7)), the band gap (*E*_g_) was estimated by extrapolating the tangent line in the plot of (*αhν*)^2^ versus *hν* (Inset in Figure 7a).

(*αhν*)^2^ = *A*(*hν* − *E*_g_) (7)

where *α*, *hν*, *A* and *E*_g_ are absorption coefficient, photon energy, the characteristic of the material and band gap, respectively. The band gap of ZnO-X was less than ZW (Table 2). All ZnO-X samples obtained by annealing ZnO-H with melamine exhibited a slight red shift to a longer wavelength compared to ZW.

To further investigate the band structure of ZnO-X and ZW, the Urbach energy (*E*_u_) was calculated according to Equation (8) [15].

ln(*α*) = *α*_0_ + *hν*/*E*_u_ (8)

where *α*, *hν*, *α*_0_ and *E*_u_ are absorption coefficient, photon energy, a constant and Urbach energy, respectively. The *E*_u_ values of samples were determined by the slope of the linear portion of ln(*α*)~*hν* curves (Figure S2 and Table 2). Compared to ZW, the higher *E*_u_ values for ZnO-X indicated that the melamine-assisted thermal activation method favors the formation of defects in ZnO, which is expected to improve the photocatalytic activity.

According to the XPS valence band (VB) spectra (Figure 7b), the VB positions of ZW and ZnO-1.2 were 2.61 eV and 2.54 eV. The band gaps of ZW and ZnO-1.2 were 3.10 eV and 3.05 eV. Thus, the conduction band (CB) position of ZW and ZnO-1.2 can be calculated by *E*_vb_ = *E*_cb_ + *E*_g_, where *E*_vb_, *E*_cb_ and *E*_g_ are VB energy, CB energy and band gap, respectively. The values of *E*_cb_ were −0.49 eV and −0.51 eV for ZW and ZnO-1.2.

### 2.4. Mechanism of Oriented-Growth and Vacancy-Rich ZnO

**Figure 8:**
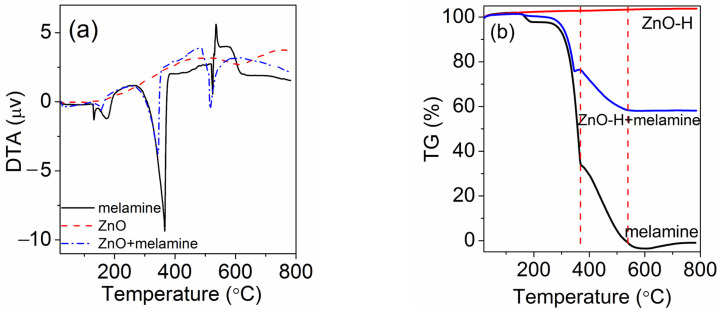
(**a**) DTA curves and (**b**) TG curves of ZnO-H, the mixture of ZnO-H and melamine (the mass ratio of ZnO to melamine was 1.2:1) and melamine.

DTA/TG analysis was employed to investigate the thermal decomposition of ZnO with and without melamine (Figure 8). Compared to pure ZnO-H and melamine, the mixture of ZnO-H and melamine showed different thermal behavior. There were four endothermic peaks (130 °C, 176 °C, 365 °C and 523 °C) and an exothermic peak at 537 °C in DTA for melamine (Figure 8a). The weight decreased dramatically in the range between 300 °C and 550 °C (Figure 8b), which was ascribed to the oxidation and decomposition of melamine. Complete decomposition of melamine at 600 °C was observed. For ZnO, no distinct peak and weight loss were measured in DTA and TG curves. The DTA curve for the mixture of ZnO-H and melamine also showed four endothermic peaks (133 °C, 154 °C, 343 °C and 516 °C), which moved to a lower temperature compared to melamine. An exothermic peak at 487 °C in the DTA curve was observed. The corresponding weight loss was measured to be 41.8%. When the temperature reached 540 °C, the weight of the mixture remained unchanged, and no weight loss occurred beyond this temperature. According to the mass ratio of ZnO to melamine (1.2:1), the percentage of melamine in the mixture was 45.5%. Combined with the XPS analyses, the weight loss (41.8%) of melamine indicated that most of melamine in the presence of ZnO decomposed and only a few carbon residues were left.

Melamine decomposed to form NH_3_ as the temperature increased. Under this environment, oxygen vacancies were easier to form in ZnO-X than in ZW under oxygen-rich conditions, which was consistent with the results from XPS and Raman analyses. The presence of melamine resulted in ZnO showing different thermal behavior and growing along a specific direction, thus enhancing the expression of (100) planes on ZnO rods, which was affected by the ratio of ZnO to melamine. Therefore, calcining ZnO with melamine provided favorable conditions for the growth of (100) planes and vacancy-induced defects. It is known that ZnO shows a strong face-dependent photocatalytic activity. Among these lattice planes, the (100) planes of ZnO showed the highest activity, while the (00-1) and (110) planes showed lower activity. According to XRD analyses, more (100) planes were obtained for ZnO-1.2, which also increased the photocatalytic activity [22].

**Figure 9:**
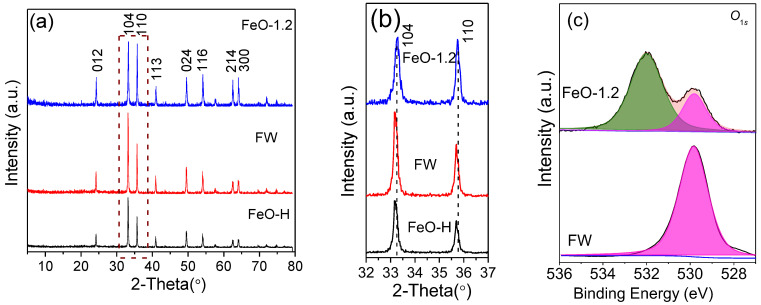
(**a**) XRD patterns and (**b**) the enlarged XRD patterns (in the region of 2θ = 32–37°) of FeO-H, FW and FeO-1.2. (**c**) The high-resolution XPS spectra of O 1*s* of FeO-H and FW.

A similar effect of melamine on the growth of α-Fe_2_O_3_ was also observed. After calcining FeO-H (α-Fe_2_O_3_ prepared by the hydrothermal method) with and without melamine (labeled as sample FeO-1.2 and sample FW, respectively), both samples showed the rhombohedral structure of α-Fe_2_O_3_ (JCPDS No. 33-0664) and no diffraction peaks for g-C_3_N_4_ were detected (Figure 9a). The calcination led to an increase in the crystallinity of α-Fe_2_O_3_. The magnified XRD patterns (Figure 9b) revealed the shift of diffraction peaks and the relatively high intensity of (110) peaks in the sample FeO-1.2 compared with FW and FeO-H, indicating that the addition of melamine changed the growth habit of α-Fe_2_O_3_. The high resolution XPS spectrum of O 1*s* for FeO-1.2 was fitted with two peaks, the O_L_ peak and O_V_ peak, which were located at 529.80 eV and 531.51 eV (Figure 9c). The XPS spectrum for FW was only fitted with one peak at 529.82 eV (O_L_), which confirmed the formation of oxygen vacancies for FeO-1.2, similar with the analyses of ZnO.

### 2.5. Photodegradation Activities

**Figure 10:**
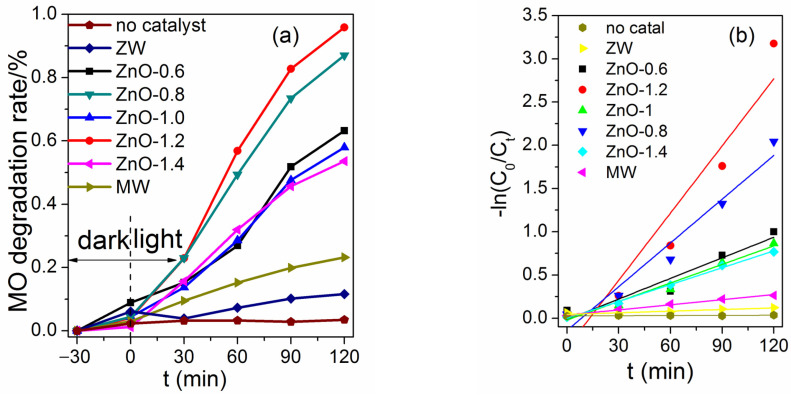
(**a**) Photocatalytic activity for ZW, MW and ZnO-X; (**b**) the first-order reaction kinetics.

The UV-Vis spectra of methyl orange (MO) after different irradiation times over ZnO-X are shown in Figure S3. The concentration of MO decreased with the extension of irradiation time. The photocatalytic activity of samples was evaluated (C_0_ and C*_t_* are the equilibrium concentrations of MO before and after irradiation, respectively). ZnO-X showed an improved photocatalytic activity. When using ZnO-0.6, ZnO-1.0 or ZnO-1.4 as the photocatalyst, the degradation rate was less than 60% (Figure 10a). When using ZnO-0.8 as the photocatalyst, the degradation rate reached 87%. It is noteworthy that ZnO-1.2 exhibited a much higher activity (95.8%) than the other ZnO-X, and the MO solution was decolorized completely after 120 min of irradiation, which was similar to Ni-doped ZnO (94.47% for dye degradation) [23], while slightly lower than composites, for example, ZnTi-LDHs/g-C_3_N_4_ (~100% for dye degradation) [24] and BiOBr/Bi_2_Sn_2_O_7_ (99.6% for dye degradation) [25]. A blank experiment in the absence of photocatalysts but under irradiation showed that a small quantity of MO was reduced. When using ZW or MW as the photocatalyst, only a small amount of MO was degraded (13% for ZW and 25% for MW after 150 min of irradiation).

The half degradation time *t*_1/2_ were used to evaluate the catalyst activity [26]. The *t*_1/2_ was 88 min, 62 min, 96 min, 53 min and 107 min for ZnO-0.6, ZnO-0.8, ZnO-1.0, ZnO-1.2 and ZnO-1.4, respectively. For MW and ZW, the low degradation rate (less than 50%) after irradiation of 120 min showed that their *t*_1/2_ values were higher than 120 min. Figure 10b shows the first order dynamic curves between −ln(*C_t_*/*C*_0_) and *t* (Table S1). The rate constants (*k*) for ZnO-X and ZW were obtained from the slope (Table 3). Among these samples, ZnO-1.2 exhibited the highest *k* value (0.0263 min^−1^), which was 43 times higher than that of ZW (0.0006 min^−1^). The order of *k* values of different ZnO/melamine ratios was 0.0263 min^−1^ (1.2:1) > 0.0169 min^−1^ (0.8:1) > 0.0079 min^−1^~0.0065 min^−1^ (0.6:1, 1.0:1, 1.4:1). It is known that ZnO with more non-polar (100) planes could exhibit better photocatalytic properties [11,22]. XRD analyses demonstrated that the oriented growth of ZnO rods in ZnO-X varied according to different ZnO/melamine ratios (Figure 1 and Figure S1). Compared with ZnO-1.2 and ZnO-0.8, the relative peak intensity of (100) planes for ZnO-1.4, ZnO-1.0 and ZnO-0.6 were lower, which resulted in the low *k* value. The lower *t*_1/2_ and the higher *k* values confirmed that ZnO-1.2 has the best photocatalytic efficiency.

**Figure 11:**
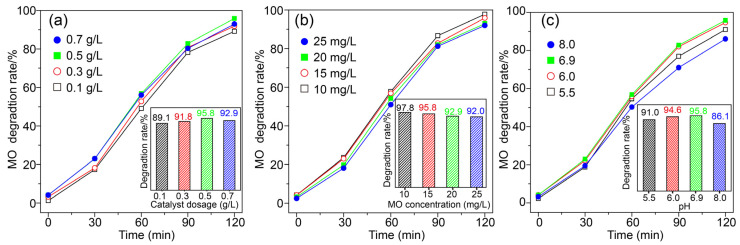
The effect of (**a**) catalyst dosage, (**b**) initial concentration of MO and (**c**) pH on the photocatalytic degradation of MO.

The effect of catalyst dosage, initial concentration of MO solution and the pH value on the photocatalytic degradation of MO were investigated (Figure 11). With the increase in catalyst dosage from 0.1 to 0.5 g/L, the degradation rate of MO increased from 89.1% to 95.8% (Figure 11a). It is known that, the more catalyst is added, more active sites are available for MO degradation, which can improve the photocatalytic activity. However, increasing the catalyst dosage further (0.7 g/L) caused a decrease in the degradation rate of MO (92.9%). Excessive catalyst would scatter the light that penetrates into the reaction system, leading to a decrease in the photocatalytic activity. Thus, an optimum catalyst dosage for the degradation of MO was 0.5 g/L. Figure 11b shows that the degradation rate of MO gradually decreased from 97.8% to 92.0% as the initial concentration of MO increased from 10 mg/L to 25 mg/L. At lower concentrations, all MO in solution could interact with the active sites on the surface of catalyst, which led to an increase in the degradation rate compared with those at higher initial MO concentrations.

The influence of initial pH of the reaction solution on the degradation of MO was investigated (Figure 11c). When the pH value increased from 5.5 to 6.9, the degradation rate of MO increased from 91% to 95.8% and then slightly decreased to 86.1% at pH 8.0. MO changes to a negatively charged azo structure when the pH value is greater than 4.5 [27]. In our experimental conditions, the pH values were between 5.5–8.0 and MO molecules were negatively charged. Photocatalytic activity is related to the formation of reactive species such as hydroxyl radical (•OH), superoxide radical anion (•O_2_^−^) and holes during the photocatalytic process. In basic conditions, it is easy for photogenerated holes to react with OH^−^ to form •OH. However, in our experiments, •OH was not the dominant active substance during the degradation process. Therefore, a basic condition has less effect on improving photocatalytic activity. With the increasing pH value, more OH^−^ in the solution would increase the competition with negatively charged MO, which could hinder the contact of MO with the catalyst and reduce the photocatalytic activity. Herein, the degradation rate of MO decreased to 86.1% while pH value increased to 8.0.

**Figure 12:**
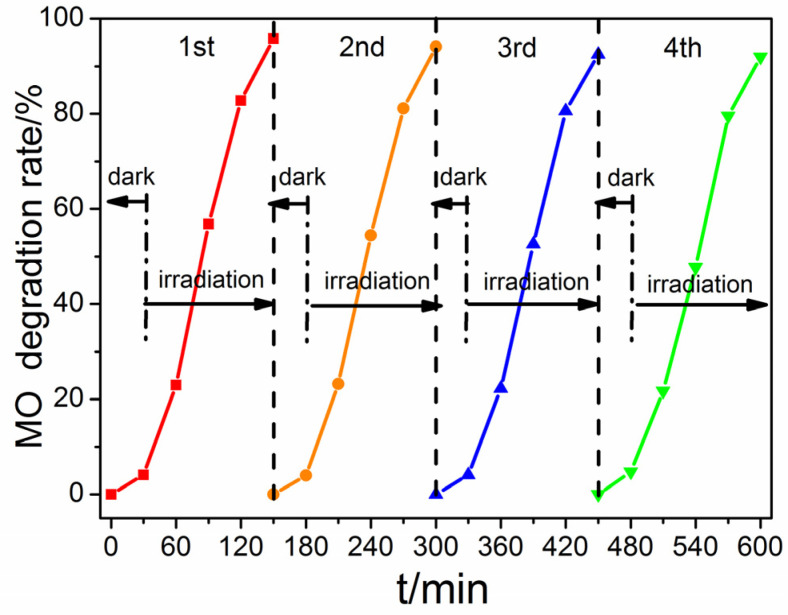
The stability of ZnO-1.2 after 4 cycles of photocatalytic degradation of MO.

To investigate the stability of ZnO-1.2, the renewability experiment was carried out. After four-cycle experiments, the degradation rate of ZnO-1.2 on MO was maintained at 92% after 120 min of irradiation (Figure 12). The reuse examination showed that ZnO-1.2 could be recycled four times without loss of degradation efficiency, which confirmed that ZnO-1.2 has good stability and regeneration capacity.

**Figure 13:**
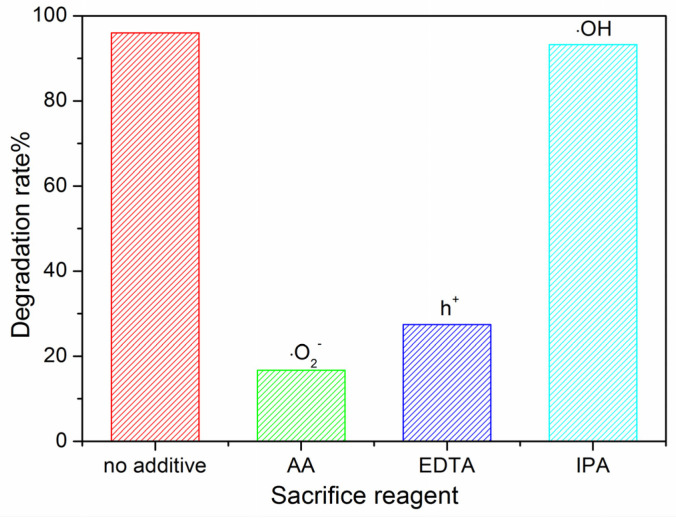
Reactive-species-trapping experiments.

To investigate the photocatalytic mechanism, reactive-species-trapping experiments were performed by adding IPA, EDTA and AA to capture the active substances •OH, h^+^ and •O_2_^−^, respectively. With the addition of AA and EDTA, the degradation rates decreased distinctly (Figure 13), which showed that •O_2_^−^ and h^+^ were the dominant active substances during the degradation process.

**Figure 14:**
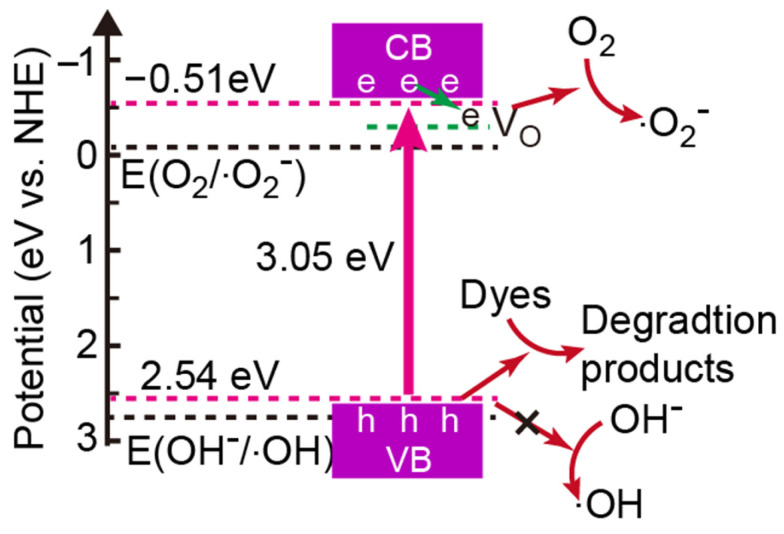
The degradation mechanism of ZnO-X.

It is known that photoinduced electrons and holes can migrate to the surface of ZnO to form superoxide and hydroxyl radicals with dissolved O_2_ and OH^−^, respectively (Equations (9) and (10)) [28]. Since the bottom of the conduction band of ZnO is less than the standard redox potential of O_2_/•O_2_^−^ (−0.31 eV), photogenerated electrons can react with O_2_ to form •O_2_^−^ (Figure 14). However, the top of the valence band of ZnO is greater than that of OH^−^/•OH (2.74 eV), meaning photogenerated holes cannot react with OH^−^ to form •OH. Therefore, the degradation rate changed slightly with the addition of IPA (•OH trapping agent). The addition of hole-trapping agent (EDTA) leading to a decrease in the degradation rates demonstrates that photogenerated holes could oxidize the pollutant. So, the reactive species during the photocatalytic process for both ZW and ZnO-1.2 are holes and •O_2_^−^.

O_2_ + ZnO(e^−^) → •O_2_^−^ (9)

OH^−^ + ZnO(h^+^) → •OH (10)

However, in our experiments, ZnO-X exhibited better photocatalytic properties in comparison with ZW. Based on the above XRD analyses, we found that calcining ZnO with melamine provided favorable conditions for the growth of non-polar (100) planes. It is known that oxygen vacancies formed mainly at the (100) surface of ZnO [29]. Combined with XPS, Raman and UV-Vis absorbance analyses, these results revealed that ZnO-X has more defects located at interfaces than ZW, which could provide more active sites for photocatalytic reaction and facilitate the transfer of photoinduced electron/hole pairs to the interface, leading to a higher photocatalytic activity of ZnO-X in comparison with ZW [29,30]. Therefore, with the enhanced adsorption properties of ZnO-X, the combination of oxygen vacancies with orientated growth led to the improvement of the photocatalytic property of ZnO-X.

## 3. Materials and Methods

### 3.1. Materials

Melamine, zinc nitrate (Zn(NO_3_)_2_·6H_2_O), sodium hydroxide (NaOH), iron chloride (FeCl_3_·6H_2_O), cetyltrimethyl ammonium bromide (CTAB), methyl orange (MO), isopropyl alcohol (IPA), ethylene diamine tetraacetic acid (EDTA) and ascorbic acid (AA) were purchased from Sinopharm Chemical Reagent Co., Ltd. (Shanghai, China) and used as received without further purification.

### 3.2. Synthesis of ZnO

Preparation of ZnO by hydrothermal method: Zn(NO_3_)_2_·6H_2_O (4.5 mmol) was dissolved in deionized water (40 mL) to form a uniform solution. Then, 60 mmol of NaOH was added to the above solution. The final concentration of Zn^2+^ and OH^−^ in the solution were 0.113 mol/L and 1.500 mol/L. After stirring for 30 min at room temperature, a white suspension was formed. Then, the suspension was transferred to a 50 mL autoclave and heated at 120 °C for 10 h without stirring. After cooling down to room temperature, the product was centrifuged and washed with deionized water and ethanol twice. The collected sample was dried at 50 °C for 2 h and labeled as ZnO-H.

Modification of ZnO by melamine-assisted thermal activation: 200 mg of ZnO-H and 200 mg of melamine were mixed evenly and annealed at 520 °C for 4 h at a heating rate of 5 °C/min. The mass ratio of ZnO-H to melamine was 1:1. The mass ratio was also modified to 0.6:1, 0.8:1, 1.2:1 and 1.4:1 by changing the amount of melamine and maintaining the amount of ZnO-H at 200 mg. After cooling, a light yellow product was obtained and labeled as ZnO-X (X refers to the ratio of ZnO-H to melamine, that is, 0.6, 0.8, 1.0, 1.2 and 1.4, respectively).

For comparison, 200 mg of melamine was annealed under the same experimental conditions but without ZnO-H. The yellow product was labeled as MW. An amount of 200 mg of ZnO-H was annealed under the same experimental conditions but without melamine. The white sample was labeled as ZW.

### 3.3. Synthesis of α-Fe_2_O_3_

This method was also used to prepare vacancy-rich α-Fe_2_O_3_. Firstly, α-Fe_2_O_3_ was obtained under the same experimental conditions as sample ZnO-H except FeCl_3_·6H_2_O (0.1 mol/L) and CTAB (0.07 mol/L) were dissolved in 40 mL of deionized water and reacted at 180 °C for 3 h. The sample was labeled as FeO-H. Secondly, a mixture of 200 mg of FeO-H and 167 mg of melamine was annealed at 520 °C for 4 h at a heating rate of 5 °C /min. The mass ratio of FeO-H to melamine was 1.2:1. After cooling, the sample was labeled as FeO-1.2. FeO-H (200 mg) was also annealed under the same experimental conditions but without melamine. The sample was labeled as FW.

### 3.4. Photocatalytic Property Measurement

Samples (0.5 g/L) were added to an MO solution (15 mg/L) and kept in the dark for 30 min to ensure adsorption–desorption equilibrium. Then, the suspension was irradiated under UV light. The pH value of the solution was about 6.9. Samples were removed from the solution after different irradiation times and analyzed using a UV-Vis spectrophotometer at 464 nm.

The effects of catalyst dosage (0.1, 0.3, 0.5 and 0.7 g/L), MO concentration (10, 15, 20 and 25 mg/L) and pH value (5.5, 6.0, 6.9 and 8.0) on the photocatalytic degradation of MO were investigated. During these processes, only one factor was changed and the other experimental conditions were the same as the above photocatalytic processes.

Renewability experiment: The renewability experiment was carried out by repeating photocatalytic degradation of MO for four cycles. After each cycle, ZnO-1.2 was collected from solution and washed three times with water for the next photocatalytic experiment. Before being illuminated, the suspension was kept in the dark for 30 min to ensure adsorption–desorption equilibrium.

Species-trapping experiments: IPA, EDTA and AA were added to the reaction solution to capture active substances •OH, h^+^ and •O_2_^−^, respectively. The concentration of trapping agents was 0.5 mmol/L.

### 3.5. Characterization

X-ray powder diffraction (XRD) patterns were recorded using a D8 ADVANCE X-ray diffractometer (Karlsruhe, Germany) with Cu Kα radiation (*λ* = 0.154 nm). The scanning electron microscopy (SEM) images were recorded on an FEI-Sirion 200F field emission scanning electron microscope (Hongkong, China). The transmission electron microscopy (TEM) image, selected area electron diffraction (SAED) patterns and the energy-dispersive spectroscopy (EDS) spectra were taken with a JEM-2100F transmission electron microscope. The diffuse reflectance spectra were recorded by a UV-Vis spectrophotometer (UV-3900H, Hitachi, Tokyo, Japan). The chemical states were investigated by X-ray photoelectron spectroscopy (XPS) on an ESCALAB 250Xi photoelectron spectrometer with Al K (1486 eV) as the excitation light source (Waltham, MA, USA). Raman spectra were investigated by a Raman microspectrometer (WJGS-034, Horiba, Paris, France) operating with an excitation laser wavelength of 532 nm. Thermogravimetric analysis (TG) and differential thermal analysis (DTA) were carried out on a HCT-4 thermal analyzer with a heating rate of 10 °C/min in air (Henven, Beijing, China). Photocatalytic reactions were carried out in an XPA-7-type photochemical reactor (Xujiang electromechanical plant, Nanjing, China) using a 300 W medium-pressure mercury lamp (mean wavelength 365 nm). The distance between the light source and the reactant was 15 cm.

## 4. Conclusions

ZnO rods with oxygen vacancies and (100) planes were successfully synthesized by a melamine-assisted thermal activation method. Simply calcining ZnO with melamine introduced oxygen vacancies in ZnO and realized the orientation growth of ZnO rods. Raman and XPS spectra revealed the formation of oxygen vacancies in the ZnO. XRD analyses demonstrated that the higher ratio of ZnO to melamine led to greater amounts of (100) planes and oxygen vacancies in ZnO, and it reached the highest when the ratio was 1.2:1. Compared with ZnO-H and ZW, ZnO-X showed improved photocatalytic activity and ZnO-1.2 exhibited the highest photocatalytic degradation rate of MO (95.8%). After four cycles of photocatalytic degradation of MO, the degradation rate was still at 92%, which confirmed that ZnO-1.2 has good stability and regeneration capacity. The reactive species during the photocatalysis reaction were holes and •O_2_^−^ for both ZW and ZnO-X. The enhancement in photocatalytic activity could be attributed to the presence of oxygen vacancies and (100) planes, which favored photoinduced charges’ separation and transfer. The more oxygen vacancies and (100) planes in the structure, the higher photocatalytic activity ZnO has. Vacancy-rich α-Fe_2_O_3_ was also prepared by this melamine-assisted thermal activation method. Our study proved that the melamine-assisted thermal activation method can simplify the preparation process of materials with defects and improve photocatalytic properties, which provides a feasible route for the future design and synthesis of photocatalysts.

## Figures and Tables

**Table 1:** Structural parameters of ZnO-H, ZW and ZnO-X.

Sample	*a* (nm)	*c* (nm)	*V* (nm^3^)	D (nm)		*ε* (×10^−4^)
				Scherrer’s Formula	W-H Model	W-H Model
ZnO-H	0.3258	0.5217	0.04796	78.5	99.0	6.5847
ZW	0.3254	0.5214	0.04781	87.1	123.8	5.4779
ZnO-0.6	0.3253	0.5212	0.04776	88.1	139.3	4.0681
ZnO-0.8	0.3251	0.5206	0.04765	88.5	133.3	3.8954
ZnO-1.0	0.3249	0.5198	0.04752	89.0	118.5	3.2812
ZnO-1.2	0.3246	0.5196	0.04741	89.6	117.5	3.2042
ZnO-1.4	0.3245	0.5194	0.04736	89.8	117.4	3.1872

**Table 2:** Calculated values of *E*_g_ and *E*_u_ for ZnO-X and ZW.

Sample	*E*_g_ (eV)	*E*_u_ (eV)
ZnO-0.6	3.08	0.236
ZnO-0.8	3.06	0.249
ZnO-1	3.07	0.223
ZnO-1.2	3.05	0.262
ZnO-1.4	3.07	0.167
ZW	3.10	0.159

**Table 3:** The kinetic parameters of MW, ZW and ZnO-X.

	MW	ZW	ZnO-0.6	ZnO-0.8	ZnO-1.0	ZnO-1.2	ZnO-1.4
*K* (min^−1^)	0.0019	0.0006	0.0079	0.0169	0.0071	0.0263	0.0065
*R* ^2^	0.9881	0.9770	0.9280	0.9452	0.9631	0.9645	0.9946

## Data Availability

Not applicable.

## Supplementary Materials

The following supporting information can be downloaded at: https://www.mdpi.com/article/10.3390/molecules28145329/s1, Equation S1: Bragg equation. Equation S2: the lattice spacing calculating equation for hexagonal structure. Table S1: −ln(*C*_0_/*C*_t_) for all samples. Figure S1: The ratio of diffraction peak intensity and the lattice parameter (a and c) of ZnO calculated from XRD patterns. Figure S2: Plots of lnα versus *hv* for ZW and ZnO-X. Figure S3: UV-Vis spectra of MO after different irradiation times over different photocatalysts (a) ZnO-0.6, (b) ZnO-0.8, (c) ZnO-1.0, (d) ZnO-1.2 and (e) ZnO-1.4.

## Sample Availability

Samples of the compounds ZnO and α-Fe_2_O_3_ are available from the authors.
