# Supplementary material for: Melamine-Assisted Thermal Activation Method for Vacancy-Rich ZnO: Calcination Effects on Microstructure and Photocatalytic Properties
Source: Molecules. 2023 Jul 11;28(14):5329. doi: 10.3390/molecules28145329 (PMC10385723; doi:10.3390/molecules28145329)
Supplement: Supplementary file 1 [file molecules-28-05329-s001.zip › molecules-2469389-supplementary.pdf]

Supporting Information for

Melamine-Assisted Thermal Activation Method for Vacancy-Rich ZnO: Calcination Effects on Microstructure and Photocatalytic Properties

Weiwei Wang [1,*], Lin Lv [2], Changfeng Wang [2] and Jiao Li [1,*]

1 School of Materials Science and Engineering, Shandong University of Technology, Zibo 255049, China
2 Shandong LinJia New Material Technology Co., Ltd., Zibo 255049, China; lyulincn@foxmail.com (L.L.); 13884621118@163.com (C.W.)
* Correspondence: wangweiwei@sdut.edu.cn (W.W.); haiyan9943@163.com (J.L.)

Equations 1 and 2 were derived from Bragg equation (Equation S1) and the lattice spacing calculating equation for hexagonal structure (Equation S2) using the lattice spacing values of (100) and (002) planes.

$$2d\sin\theta=\lambda \quad \text{(S1)}$$

$$d^{-1}=[4(h^2+hk+k^2)/3a^2+l^2/c^2]^{1/2} \quad \text{(S2)}$$

Table S1. -ln(C_0/C_t) for all samples

Illumination time (min)	-ln(C_0/C_t)							
	ZnO-0.6	ZnO-1.2	ZnO-1	ZnO-0.8	ZnO-1.4	CN	ZnO	no catal
0	0.0932	0.0419	0.0450	0.0450	0.01207	0.0305	0.0630	0.0232
30	0.1656	0.2606	0.1474	0.2615	0.1702	0.0996	0.0397	0.0323
60	0.3133	0.8400	0.3363	0.6807	0.3850	0.1652	0.0756	0.0329
90	0.7300	1.7600	0.6462	1.3254	0.6101	0.2221	0.1069	0.0290
120	1.0004	3.1770	0.8663	2.0425	0.7677	0.2642	0.1231	0.0356

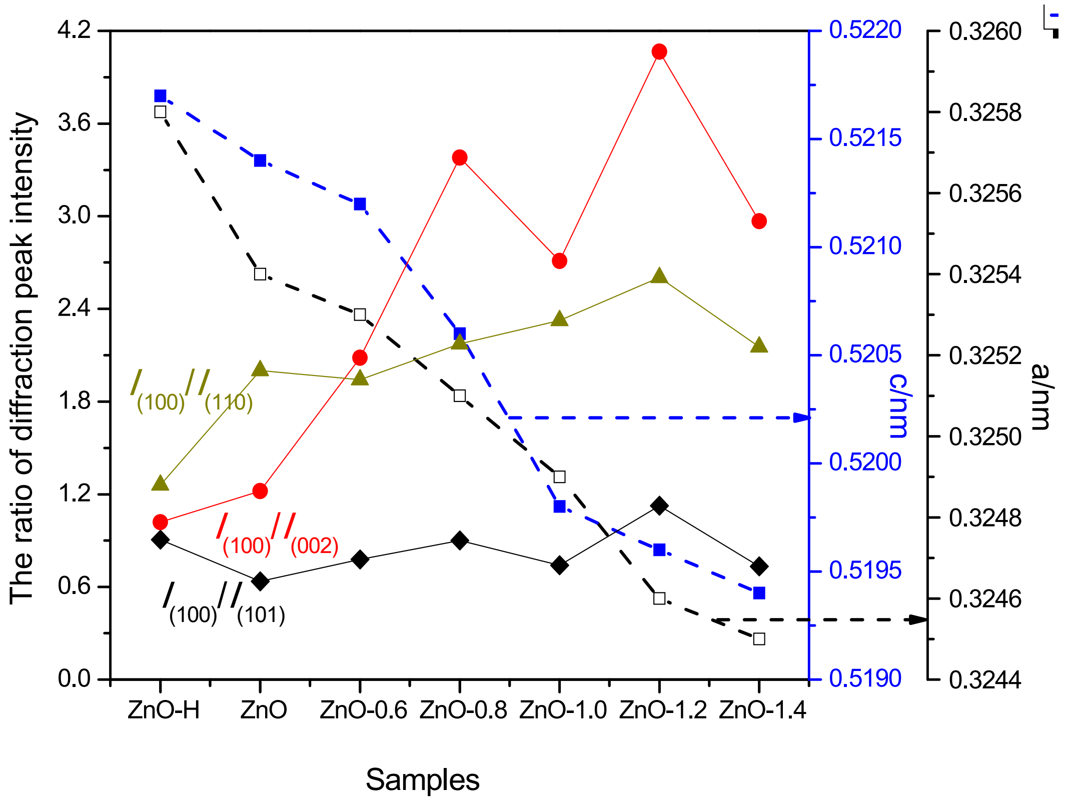

Figure S1. The ratio of diffraction peak intensity and the lattice parameter (*a* and *c*) of ZnO calculated from XRD patterns.

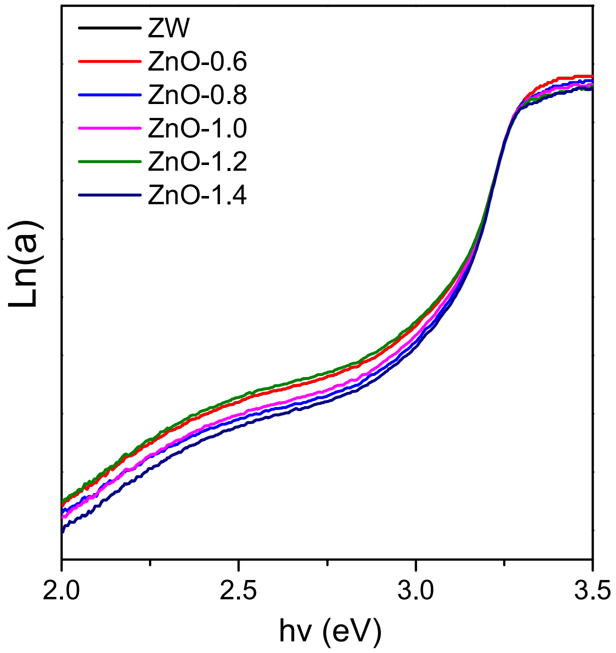

Figure S2. Plots of ln*α* versus *hv* for ZW and ZnO-X.

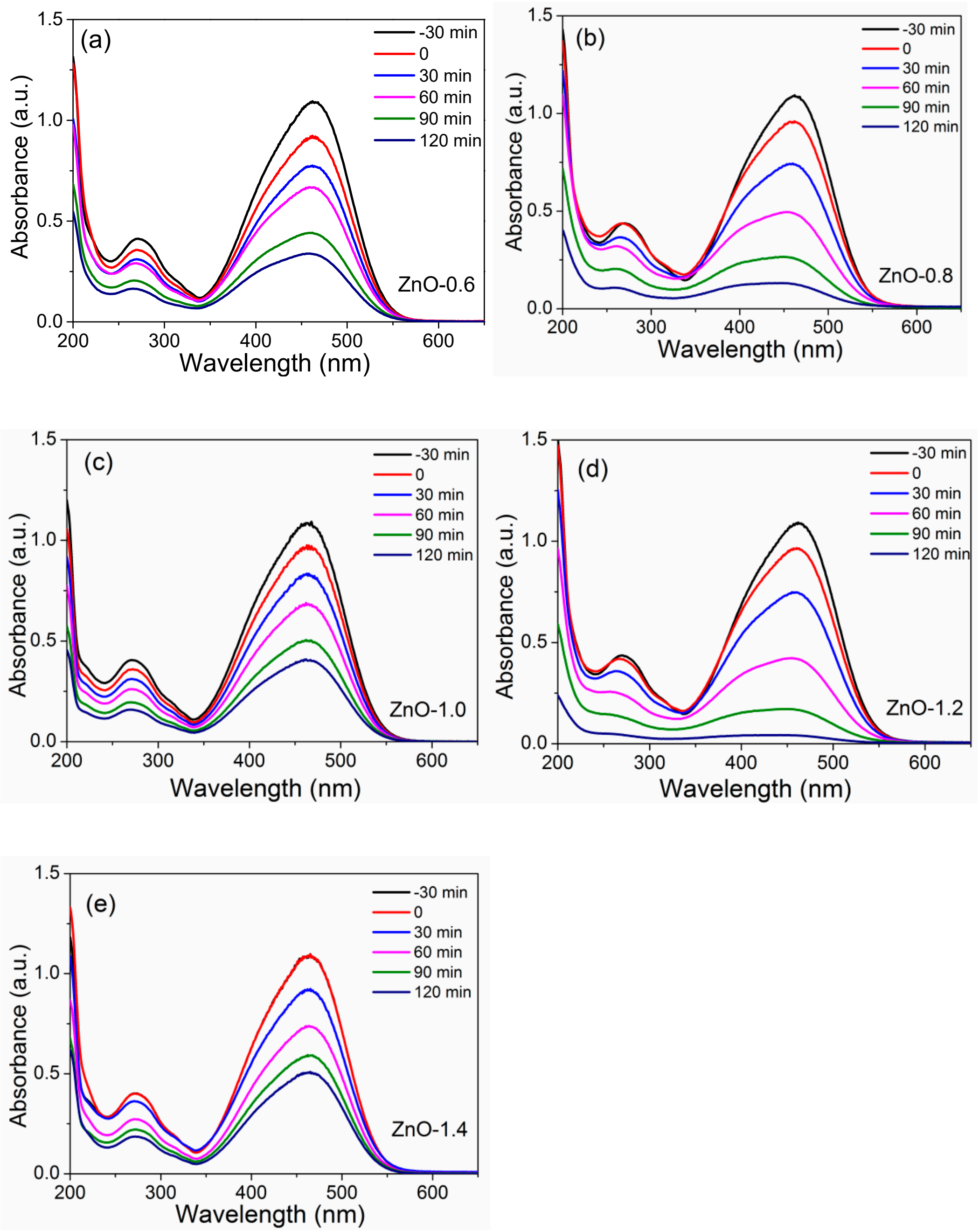

Figure S3. UV-vis spectra of MO after different irradiation times over different photocatalysts (a) ZnO-0.6, (b)ZnO-0.8, (c)ZnO-1.0, (d)ZnO-1.2, and (e)ZnO-1.4
